# Prevalence and trends of cervical cancer screening among women in Fiji from 2014 to 2018

**DOI:** 10.1016/j.heliyon.2024.e30220

**Published:** 2024-04-26

**Authors:** Aliti Kunatoga, Masoud Mohammadnezhad, Sabiha Khan, Parisa Naeem, Pushpa Nusair

**Affiliations:** a School of Public Health and Primary Health Care, Fiji National University, Suva, Fiji; b School of Nursing and Midwifery, Birmingham City University, Birmingham, UK; c Oceania Hospitals, Suva, Fiji; d School of Life Sciences, University of Bradford, Bradford, UK

**Keywords:** Cervical cancer, Prevalence, Cancer screening, Trends, Pap smear, Fiji

## Abstract

**Introduction:**

Cervical cancer is the third most common cancer in women both in developed and developing countries. This study aimed to determine the prevalence of cervical cancer and the trends of cervical cancer screening among women who had cervical cancer screening in Suva, Fiji between 2014 and 2018.

**Materials and method:**

This study applied a 5-year retrospective electronic chart review of data from all women attending the Women's Wellness Clinic (WWC) in Suva, Fiji. The women who were selected for this study and screened for cervical cancer were Fijian citizens above 18 years of age and were registered in 2014–2018. A data collection form was used to collect data. The data was analyzed using Statistical Package for Social Sciences (SPSS) version 24; p <0.05 % was considered as the level of significance.

**Results:**

Among the 39,579 women who attended WWC for other family planning services, 12,074 women screened for cervical cancer with a prevalence of 30.5 %. The overall mean age for women screened for cervical cancer was 37.6 (SD ± 11.2). Two-thirds (76.4 %) of the women screened for cervical cancer were less than 46 years of age and 53.9 % were I-taukei. The number of women who came for Pap smear increased in 2015, however, a slight decline was observed in 2016 which was later improved to 35.1 % in 2018. Malignancy was more common in the age range of 42–49 respectively. In this study, women of 46 years and above had an OR of 0.51 (95 % CI: 0.36, 0.72), other ethnicity OR was 1.73 (95 % CI: 1.27, 2.35), and the Muslim religion OR recorded was 1.44 (95 % CI: 1.03, 2.01) which was comparatively considered a high-risk group. Women who are widowed 1.57 (95 % CI: 0.798, 3.11), single 1.29 (95 % CI: 0.87, 1.92) or divorced 1.08 (95 % CI: 0.59, 1.99), employed 1.01 (95 % CI: 0.83, 1.24) and are living in rural areas 1.19 (95 % CI: 0.82, 1.73) are also associated with higher odds of having abnormal results.

**Conclusion:**

Cervical cancer is listed as the first and most common type of cancer in women which is noticeably increasing in Fiji. Even though cervical cancer screening has improved over the years, adequate surveillance systems and ongoing programs should be designed and implemented to increase awareness and monitor the trend of cervical cancer screening in order to reduce cervical cancer prevalence and mortality rates.

## 1 Introduction

Non-communicable Diseases (NCDs) remain a significant public health concern responsible for abundant illness and deaths globally [[1], [2], [3]]. Cancer is one of the NCDs that is expected to rank as the leading cause of death, and the single most important barrier to increasing life expectancy in every country of the world in the 21st century [[4], [5], [6], [7]]. The NCDs that result from cancer are considered as the leading cause of mortality affecting life expectancy. Worldwide, people in Low and Middle-Income Countries (LMICs) are commonly targeted by this [8]. Cervical cancer is considered the fourth most commonly diagnosed neoplasia in women accounting for 604,127 cases annually [9]. It is also well established to be the third most common cancer in females with 265,700 mortality rates per year. Although, cancer incidence and mortality rates vary geographically, nevertheless, most cancers and associated deaths occur in less-developed regions [4,[10], [11], [12], [13]].

Cervical cancer is categorized into 6 stages namely Atypical Squamous Cells of Undetermined Significance (ASCUS), Possible Low-Grade Squamous Intraepithelial Lesion (PLGSIL), Low-Grade Squamous Intraepithelial Lesion (LGSIL), Possible High-Grade Squamous Intraepithelial Lesion (PHGSIL), High Grade Squamous Intraepithelial Lesion (HGSIL) and Malignant stage [9,14]. As indicated, some of the cells on the Pap smear do not entirely appear normal but also do not meet the diagnostic criteria for a lesion [15]. PLGSIL refers to the possibility of having a low grade result, LGSIL occurs when abnormal cells start to form on the surface of certain organs like the cervix [[16], [17], [18], [19]]. PHGSIL illustrates the possibility of having high grade result, HGSIL occurs when the cells appear dysplastic under a microscope. They are usually caused by chronic infection with certain types of Human Papillomavirus (HPV) and are found when a Pap test or biopsy is done [16,[20], [21], [22]] and Malignancy is when the cells grow and spreads to other parts of the body [23,24]. Risk factors for cervical cancer mainly include chronic infection with certain types of Human papillomavirus (HPV) that are routinely detected by Pap test or biopsy [1,2,11] [[1], [2], [11], [12], [13], [14], [15], [3], [4], [5], [6], [7], [8], [9], [10]] [3,15] [[1], [2], [11], [12], [13], [14], [15], [3], [4], [5], [6], [7], [8], [9], [10]] [[1], [2], [11], [12], [13], [14], [15], [3], [4], [5], [6], [7], [8], [9], [10]]. Malignancy occurs when the neoplastic cells spread to the surrounding tissues and organs.

Within the last decade cervical cancer has been indicated to be the most common cancer and the leading cause of mortality among women of Fiji. Population. Figures show cervical cancer incidence is higher in Fiji (51 per 100,000), Tonga (16 per 100,000), Cook Islands (17 per 100,000) and Niue (26 per 100,000) compared to Pacific women in New Zealand (11 per 100,000) or New Zealand in general (8 per 100,000) [[25], [26], [27], [28], [29]]. In 1996, the thin Prep Pap test was introduced which further increased screening success. The ThinPrep, liquid-based cervical cytology was developed to improve sensitivity by providing a monolayer of cells to the cytologist for review. Henceforth improving diagnostic reliability of Papanicolau (Pap) smears in Fiji [16]. Despite the high prevalence rate of this cancer, cytology or pap tests are routinely recommended to women in Fiji and other countries every three years, since 2003 [30]. These tests and follow-ups have immensely decreased cancer progression and death rates in women, especially in those with abnormal test results. Even though new cases of cervical cancer and deaths have decreased since the HPV vaccine and cervical screening programs were introduced, cervical cancer still remains prevalent with a good prognosis if detected in a timely manner [19,31,32].

Although there has been remarkable advancement in terms of various effective cervical cancer screening strategies and preventions worldwide. Nevertheless, significant gaps still prevail that prevent the access of Pap smear and cervical screening for women in Fiji. Most women are still unaware of the risk factors associated with cervical cancer and the precautionary steps taken to prevent it. This study aims to determine the prevalence of cervical cancer and the trends of cervical cancer screening among women who had cervical cancer screening at the Women's Wellness Clinic (WWC) in Suva, between 2014 and 2018.

## 2 Methods

### 2.1 Study design and setting

This quantitative study used a 5-year retrospective folder audit from the years 2014–2018 at WWC in Suva, Fiji. The WWC is the central hub for areas of women's health. It is located right next to the Colonial War Memorial Hospital (CWMH) where it provides great service to women and is the largest government run reproductive health facility for the central and Eastern division of Fiji. It covers the rural and urban areas and does not have a specified number of women screened because it caters for every woman who needs to be screened. It also collaborates with Gyneclinic; where women are referred to when they are diagnosed with cancer.

### 2.2 Study sample

In this study all the Pap-smear records of women registered in the WWC within five years of service (January 1, 2014–December 31, 2018) were considered. Women screened for cervical cancer were all above 18 years of age and of Fijian origin. Those women with incomplete details were excluded in this study.

The cervical cancer-screening registry located at the WWC was used as the sampling frame. The register consisted of all the cervical-screened patients in the WWC. Patients were identified according to their hospital numbers. The folders that contained medical records of women screened for cervical cancer was securely placed and only retrieved when data was required.

All cervical-screened patients who were registered from January 1, 2014 to December 31, 2018 were retrieved and examined according to the inclusion criteria. Consecutive sampling was used to select the records/profiles of patients’ folders from the register. A total of 12,074 samples from women who were screened was selected based on the inclusion and exclusion criteria. The total records of women screened for cervical cancer registered in January 2014 to December 2018 was N = 14,502, 1554 records were excluded based on the inclusion and exclusion criteria and those with multiple or duplicated records, 874 records were excluded due to incomplete information and the total samples included in data collection & analysis was N = 12,074, respectively.

### 2.3 Data collection tool

A retrospective electronic chart review was performed on data from all women attending the cervical cancer screening services at the WWC from 2014 to 2018. In this study, a data collection form with a serial number, collection information on the folder registration number, name of the health center and National Health Number (NHN), were used. It comprised two sections. The former included the basic information such as age, address, and ethnicity which consisted of “I-Taukei” for the indigenous Fijian, Fijian of Indian Decent (FID) and Fijian of Other Descent (FOD), such as those with European, Rotuman and Chinese descent and other patients from different ethnic descent residing in Fiji, and the time the screening was performed. The latter section contained the Pap-smear test results. The dependent variable was cervical cancer screening. Independent variables were age, gender, ethnicity, geographic location, and results of screened patients (abnormality results).

### 2.4 Study procedure and data analysis

The study was conducted by the researcher retrieving information from the cervical register book and medical folders of women screened for cervical cancer during and after clinical hours to ensure that the data was collected appropriately and on time with no information missing. Patient information that was missing was retrieved from Patient Administration Information System (PATIS). Any incomplete information from the cervical cancer registration books, medical folders and PATIS was excluded to reduce biased results.

The collected data was collated and cleaned using Microsoft excel. This was then entered into International Business Machine (IBM) Statistical Package for Social Sciences (SPSS) version 24 to be analyzed and yield appropriate results. Once entered, data was cleaned to avoid inaccuracies and outliers. Continuous variables were described using the mean and standard deviation. Descriptive statistics were used to determine the stages of cervical cancer as well as the percentage distribution of each independent variable with regard to cervical cancer. Bivariate logistic regression analysis was done to determine the association of each independent variable to the dependent variable cervical cancer. Pearson chi-square test was used to test the independence of the categorical variables. P-value <0.05 was considered significant.

### 2.5 Ethical consideration

Ethics approvals for this study were granted by the Fiji National University's (FNU) College Health Research and Ethics Committee (CHREC) with the ID: 09419 and the Fiji National Health Research Ethic and Review Committee (FNHRERC). All data were fully anonymized prior to being accessed, therefore, the ethics committee waived the requirements for informed consent.

## 3 Results

### 3.1 Socio-demographic characteristics of women

Approximately two-thirds (76.4 %) of the women screened for cervical cancer were less than 46 years of age, 53.9 % were I-taukei which comprises more than half (57 %) of Fiji's total population. The results also revealed that 91.1 % were married, 64.3 % were unemployed and most lived in urban settings (93.8 %). More than half of the participants (56.6 %) were Christians and 75 % were follow-ups, depending on the results of their pap-tests (Table 1).

**Table 1:** General characteristics of women screened for cervical cancer who attended WWC between 2014 and 2018 (n = 12,074).

Characteristics	Frequency (n)	Per cent (%)
**Age in year**		
18–25	1634	13.5
26–35	4302	35.6
36–45	3299	27.3
≥46	2839	23.6
**Ethnicity**		
I- Taukei	6512	53.9
FID	4497	37.2
FOD	1065	8.9
**Marital status**		
Single	612	5.1
Married	10996	91.1
Widowed	171	1.4
Divorce	295	2.4
**Occupational status**		
Employed	4314	35.7
Unemployed	7760	64.3
**Religion**		
Christians	6831	56.6
Hindu	3702	30.7
Muslim	949	7.9
Other	592	4.8
**Residential location**		
Urban	11327	93.8
Rural	747	6.2
**Visits**		
First Visit	3023	25.0
Re-Visit	9051	75.0

## 4 The proportion of screened women

Among the 39,579 women who attended WWC for other family planning services during the study duration, 12,074 were women screened for cervical cancer with a prevalence of 30.5 %. The results illustrate that 35.1 % of women came in for screening in the year 2018 whereas only 24 % visited for screening in 2016 (Table 2).

**Table 2:** Frequency of women screened in WWC based on year.

Year	Frequency of women attending the clinic (n)	Frequency of screened women (n)	Percent (%)
**2014**	6946	2098	30
**2015**	7893	2538	32.1
**2016**	9184	2220	24
**2017**	7904	2527	31.9
**2018**	7652	2691	35.1
Total	39,579	12,074	30.5

## 5 Trends of screening based on years

**Fig. 1:**
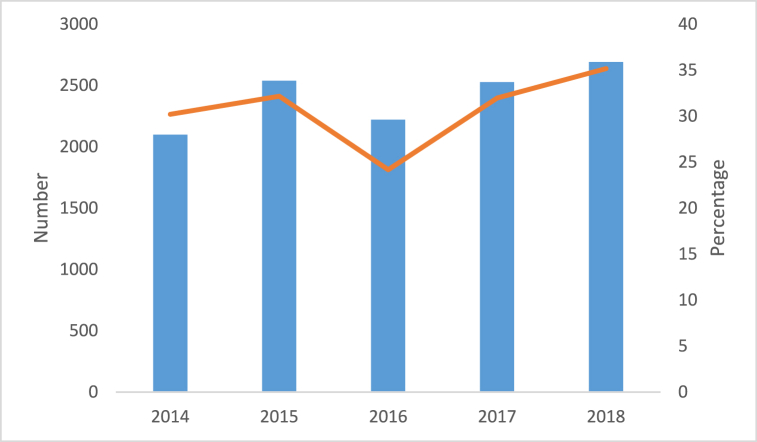
Trends of women screened for cervical cancer within the years 2014–2018.

The trends of women screened for cervical cancer can be seen in Fig. 1 where the number of women who came for Pap smear was higher in 2015 compared to 2016. Contrarily an increase in 2017 and 2018 was observed that indicate the number or the proportion of women who are attended WWC for Pap smear.

### 5.1 Age group and pap smear results

Table 3 shows the age range of women screened for cervical cancer. Most of the women who tested positive with Low-Grade Squamous Intraepithelial Lesion (LGSIL) (n = 100) were aged between 26 and 33 years. In addition, out of the total number of women screened, n = 36 tested positive for High Grade Squamous Intraepithelial Lesion (HGSIL) and were aged between 34 and 41 years. Malignancy (n = 6) in screened women seemed to be more common in the age range of 42–49 respectively. The data depicts that Atypical Squamous Cells of Undetermined Significance (ASCUS) are more common (n = 3) in women within the age group of 34–41 years whereas, PLGSIL and PHGSIL are more commonly seen (n = 40) in younger women, aged between 26 and 33 years.

**Table 3:** Age group and Pap smear results of screened women.

Age Group	Normal (n)	ASCUS (n)	LGSIL (n)	HGSIL (n)	Malignancy (n)	PLGSIL & PHGSIL (n)	Total (n)
18–25	1550	1	63	7	1	12	1634
26–33	3253	2	100	33	4	40	3432
34–41	2907	3	84	36	5	24	3059
42–49	1898	1	34	16	6	14	1969
50–57	1202	1	21	10	5	12	1251
58–65	561	0	12	2	1	2	578
66–73	130	0	0	1	0	0	131
74–90	20	0	0	0	0	0	20
**Total**	11521	8	314	105	22	104	12074

### 5.2 Association between sociodemographic characteristics and abnormal results

The results of the bivariate analysis showed that majority of the women screened for cervical cancer were within the age range of 26–35, (37.7 %) were of i-taukei ethnic group (46.8 %) married, (89 %) unemployed, (63.9 %) Christian denomination, (51.3 %) living in urban areas, (95.5 %) re-visited for follow-ups and (74.5 %) were associated with abnormal results. The bivariate analysis with age ≥46 revealed (OR = 0.51, 95 % CI: 0.36–0.72, p = 0.001), Fijian of other descents ethnicity showed (OR = 1.73, 95 % CI: 1.27–2.35, P < 0.0001) and Muslim women showed (OR = 1.44, 95 % CI: 1.03–2.01, P = 0.033) to be statistically significant of having abnormal results in addition to significantly poor cervical cancer screening (Table 4).

**Table 4:** Bivariate analysis of participant's socio-demographic characteristics on abnormal results (n = 427).

Characteristics	Normal (n)	Abnormal n (%)	P value	OR (95 % CI)
Age in year			<0.001	
18–25	1550	71 (16.6 %)	–	1
26–35	4092	161 (37.7 %)	0.296	0.85 (0.65, 1.14)
36–45	3133	131 (30.7 %)	0.545	0.91 (0.68, 1.23)
≥46	2746	64 (15.0 %)	**0.001**	0.51 (0.36, 0.72)
**Ethnicity**			**0.001**	
I- Taukei	6247	200 (46.8 %)	–	1
Indo-Fijian	4280	172 (40.3 %)	0.032	1.26 (1.02, 1.55)
Others	994	55 (12.9 %)	**<0.0001**	1.73 (1.27, 2.35)
**Marital Status**			0.361	
Married	10502	380 (89.0 %)	–	1
Single	579	27 (6.3 %)	0.213	1.29 (0.87, 1.92)
Widowed	158	9 (2.1 %)	0.191	1.57 (0.798, 3.11)
Divorce	282	11 (2.6 %)	0.810	1.08 (0.59, 1.99)
**Occupational Status**			0.918	
Unemployed	7400	273 (63.9 %)	–	1
Employed	4121	154 (36.1 %)	0.900	1.01 (0.83, 1.24)
**Religion**			0.065	
Christians	6541	219 (51.3 %)	–	1
Hindu	3528	138 (32.3 %)	0.160	1.17 (0.94, 1.45)
Muslim	892	43 (10.1 %)	**0.033**	1.44 (1.03, 2.01)
Other	560	27 (6.3 %)	0.081	1.44 (0.96, 2.17)
**Residential Location**			0.753	
Urban	10811	21 (95.5 %)	–	1
Rural	710	1 (4.5 %)	0.357	1.19 (0.82, 1.73)
**Visits**			0.830	
First Visit	2888	109 (25.5 %)	–	1
Re-visit	8633	318 (74.5 %)	0.830	0.98 (0.78, 1.22)

## 6 Discussion

This study aimed to determine the prevalence of cervical cancer and the trends of cervical cancer screening among women who had cervical cancer screening at WWC in Suva, between 2014 and 2018. The data of this report shows the number of women who had undergone cervical screening, the trend within the 5 years of screening and the results of the screening.

The results of this project showed a 35.1 % rate of Pap smear coverage of women screened in WWC among 7652 women who visited for other family planning services in 2018. The results were higher compared to the results of Pap smear coverage conducted in Fiji [29] which was 8.0 % (95 % CI: 7.9–8.1) of the target population, collected from all pathology laboratories, cancer and death registries in Fiji from 2004 to 2007. Our results are in accordance to a similar study conducted in China [33] where only 21 % of 51,989 women had reportedly had a Pap test, even though they used a nationwide survey with face-to-face interview and physical measurements that was performed every three years based on the China National Surveillance Points system.

In this study, the lowest number of women to get screened was in the year 2014 (30 %), this figure increased in 2015 (32.1 %), with a slight decrease in 2016 (24 %) and an incline from (31.9 %) to (35.1 %) in 2017 and 2018 respectively. This study was similar to an investigation conducted in Malawi [34] where supportive supervision, program data, quarterly and annual reports were analyzed from the National Cervical Cancer Control Program to evaluate the uptake and challenges of screening services from 2011 to 2015. It was observed that the number of women who were screened within a span of 5 years kept increasing. Several factors could have influenced the increase in cervical screening; Increased awareness in educating women about the importance of pap smear screening; Availability and accessibility of the screening services in clinics; Improvement of healthcare infrastructures such as better equipment's and better trained health care providers; Social support from families and friends may have encouraged women to get tested or provide transportation to screening locations.

Conversely, another study in Fiji, reported [29,32,35] with a different outcome in which the trends in cervical cancer instead of cervical cancer screening were identified. This was performed using case numbers, incidence rates and case fatalities over the decade 2000–2010 and it was observed that between 2000 and 2010, only 1234 patients were registered for cervical cancer screening. The study concluded that the number of women screened were very low.

Cervical cancer screening is free of cost in public hospitals and clinics in Fiji [17], compared to private hospitals. This is similar to the study conducted by the Epidemiology Working Group of the European Cervical Cancer Screening Network (ECCSN) [36] held at 35 centers in 20 European countries with reliable cervical cancer incidence and/or mortality data in databanks organized by the International Agency for Research on Cancer (IARC) and the World Health Organization (WHO). This study indicated that the screening was free of charge, but payment practices varied depending on the area or mode of screening activity.

Moreover, not all countries have a high participation rate in cervical cancer screening, a study conducted in South East-Nigeria [37] evaluated the level of participation in a highly subsidized cervical screening and it was observed that the level of participation in cervical screening was very low, as <1 % of the targeted women population participated within the 10-year period (1995–2004).

Of the 12074 women who were screened in this study, the majority (95.4 %) tested negative (normal) for cervical cancer. Similarly, 0.1 % tested positive for ASCUS, 2.6 % tested positive for LGSIL, 0.9 % tested positive for HGSIL, 0.2 % for Malignancy, and a total of 0.9 % for PLGSIL and PHGSIL respectively. The results of this study is in line with other studies that found ASCUS, LGSIL, HGSIL and malignancy showed abnormal results or outcome for cervical cancer screening [4,23,24,[38], [39], [40], [41]]. Most of the women (3253) who were negative for cancerous lesions, were in the age range of 26–33. The same results were observed in women who tested positive for LGSIL (100), PLGSIL and PHGSIL [40]. In Fiji, women who are above 18 years of age are eligible for screening. Alternatively, it is suggested that countries with different target population such as Finland [42], can only offer screening to women within the age range of 30–60 respectively.

A study conducted in the US population [43] to examine routine cervical cancer screening diagnoses and outcomes on age specific basis, found that CIN 1 (LGSIL category) incidence peaked among younger women aged 20–24 years. However, in this study women who tested positive with ASCUS [3] and HGSIL [36] were in the age range of 34–41 and those with malignancy were 42–49 years of age. In addition, this study also suggested that the abnormal test results were mostly observed in elderly women. Our data are consistent with this study [43] in which the CIN2/3 (HGSIL) were observed to be higher among those women with 25–29 years of age. The most common finding in this result was LGSIL followed by HGSIL. In a similar study conducted in Italy [44], the most frequent finding was ASCUS followed by LGSIL.

Moreover, cervical screening does not only reveal the abnormal results but also other infections associated with them. In a hospital based cross-sectional study with the aim of determining the occurrence of cervical precancerous changes and cervical microbial infections (Trichomonas vaginalis, Candida albicans, Neisseria gonorrhea and Actinomyces) among women attending Family Health Option Kenya (FHOK) clinic in Thika [45], it was found that out of the 244 women screened, 238 (97.5 %) presented with cervical inflammation, 80 (32.8 %) cervical microbial infections and 12 (4.9 %) cervical precancerous changes; 10 (83.3 %) with CIN I and 2 (16.7 %) with CIN II. Of the 80 cervical microbial infections, 62 (77.5 %) were yeast cells and 18 (22.5 %) T. vaginalis. A total of 134 (55 %) participants had no history of Pap smear screening, of which 84 (62.7 %) were 20–40 years old. In addition, the use of Intra Uterine Contraception Device (IUCDs) (OR: 2.47, 95 % CI 1.3–4.6) was also associated with cervical inflammation.

In our study, positive results were observed in the pap smear however they lacked the ability to detect other associated infections. Although, there were some possible LGSIL and HGSIL (0.9 %) findings in this study. These results however, were not classified under a categorized group of LGSIL or HGSIL respectively.

In this study, women above 46 years of age OR = 0.51 (0.36, 0.72), belong to other ethnicity groups such as Rotumans, Chinese and Pacific Islanders that are in Fiji OR = 1.73 (1.27, 2.35), Muslim women included; OR = 11.44 (1.03, 2.01) were found to be at higher risk of developing cervical cancer. Women who are widowed 1.57 (95 % CI: 0.798, 3.11), single 1.29 (95 % CI: 0.87, 1.92) or divorced 1.08 (95 % CI: 0.59, 1.99), employed 1.01 (95 % CI: 0.83, 1.24) and are living in rural areas 1.19 (95 % CI: 0.82, 1.73) are also associated with higher odds of having abnormal results.

There was no significant result on the independent variables that predicted any malignancy. Moreover, a cross sectional study with the aim of determining the socio-demographic factors associated with advanced stage of cervical cancer at diagnosis in Kenyatta National Hospital [13]found that older age (50–75 years) was one of the factors that was independently associated with advanced stage of cervical cancer. Similarly, in Italy in a recent cross-sectional study [46] with the aim of evaluating the history of Pap-smear in HIV-Positive women also examined the socio-demographic, clinical and organizational factors associated with adherence to cervical cancer screening found that the lack of Pap smear in the previous years was significantly associated with age <35years (OR = 1.4, compared to age ≥45 years) and lower awareness and literacy rates (OR = 1.3).

Furthermore, in a study conducted in Chicago [47] with the aim of assessing rates of Papanicolaou (Pap) testing interlinked with religion-related considerations among a racially and ethnically diverse sample of American Muslim women revealed that women who were not screened considered their health concerns as a punishment from God. This was supported with a study from Thailand [48] where Muslim women were put off from the test due to their fear of pain and embarrassment.

Women especially in the pacific should be educated on the Pap smear or any other health related tests. As most women still have less or no knowledge regarding these tests. Our study, focused on the associations of socio-demographic characteristics on abnormal results, however based on the reviewed literature, there were other factors that played important roles in abnormal results; these include obesity, household income, number of children, hazardous drinking and smoking.

## 7 Limitations

The results of this study cannot be generalized to all Fijians as the information of women who attended WWC in Suva were analyzed. Like other retrospective studies, the results of this study heavily depend on the availability of and might be affected due to missing and incomplete information that was unrecoverable or unrecorded properly from the patient's folders.

## 8 Conclusion

In Fiji, according to the well-established studies and ministry of health, cervical cancer is listed as the first and most common type of cancer in women which is noticeably increasing. New number of cases is higher in the central region compared to other parts of Fiji. Although, Fiji has improved compared to the previous years on the pap smear coverage, yet there is still a need for more widespread screening coverage especially in the rural areas. Therefore, more investigation on cervical cancer and its prevention, awareness programmes, stakeholder's consultations, and adequate surveillance systems should be designed and implemented given the resource restriction to increase awareness, monitor cervical screening trends to reduce cervical cancer prevalence and mortality rates.

## Consent for publication

Not applicable.

## Funding

The author(s) received no specific funding for this work.

## Ethics approval and consent to participants

Ethics approvals for this study were granted by the Fiji National University's (FNU) College Health Research and Ethics Committee (CHREC) with the ID: 09419 and the Fiji National Health Research Ethic and Review Committee (FNHRERC).

## Data availability statement

The data will be available from the corresponding author on request.

## CRediT authorship contribution statement

**Aliti Kunatoga:** Writing – review & editing, Writing – original draft, Visualization, Validation, Project administration, Methodology, Investigation, Formal analysis, Data curation, Conceptualization. **Masoud Mohammadnezhad:** Writing – review & editing, Validation, Supervision, Methodology, Formal analysis, Conceptualization. **Sabiha Khan:** Writing – review & editing, Supervision, Methodology, Formal analysis, Conceptualization. **Parisa Naeem:** Writing – review & editing, Supervision, Methodology, Conceptualization. **Pushpa Nusair:** Writing – review & editing, Supervision, Methodology, Conceptualization.

## Declaration of competing interest

The authors declare the following financial interests/personal relationships which may be considered as potential competing interests:One of the authors is an Advisory Board Member (ABM) of this journal.

## References

[1] Mugassa A.M., Frumence G. (2020).

[2] Roberts G., Tulloch J., World Health Organization (2011). Regional office for the western pacific., asia pacific observatory on health systems and policies. The Fiji Islands health system review.

[3] Obel J., McKenzie J. (2015). undefined. Mapping HPV vaccination and cervical cancer screening practice in the pacific region-strengthening national and regional cervical cancer prevention. ncbi.nlm.nih.gov. LBL… journal of cancer.

[4] Jemal A., Center M.M., Desantis C., Ward E.M. (2010). Global patterns of cancer incidence and mortality rates and trends. Cancer Epidemiol. Biomarkers Prev..

[5] Jemal A., Bray F., Center M.M., Ferlay J., Ward E., Forman D. (2011 Mar). Global cancer statistics. CA A Cancer J. Clin..

[6] Bray F., Ferlay J., Soerjomataram I., Siegel R.L., Torre L.A., Jemal A. (2018 Nov). Global cancer statistics 2018: GLOBOCAN estimates of incidence and mortality worldwide for 36 cancers in 185 countries. CA A Cancer J. Clin..

[7] Bray F., Ferlay J., Soerjomataram I., Siegel R.L., Torre L.A., Jemal A. (2018). Global cancer statistics 2018: GLOBOCAN estimates of incidence and mortality worldwide for 36 cancers in 185 countries. CA A Cancer J. Clin..

[8] Gravitt P.E., Silver M.I., Hussey H.M., Arrossi S., Huchko M., Jeronimo J. (2021). Achieving equity in cervical cancer screening in low- and middle-income countries (LMICs): strengthening health systems using a systems thinking approach. Prev. Med..

[9] Human Papillomavirus and Related Diseases Report WORLD [Internet] Available from:: www.hpvcentre.net.

[10] Touch S., Oh J.K. (2018). Knowledge, attitudes, and practices toward cervical cancer prevention among women in Kampong Speu Province, Cambodia. BMC Cancer.

[11] Small W., Bacon M.A., Bajaj A., Chuang L.T., Fisher B.J., Harkenrider M.M. (2017 Jul 1). Cervical cancer: a global health crisis. Cancer.

[12] Ginindza T.G., Sartorius B. (2018). Projected cervical Cancer incidence in Swaziland using three methods and local survey estimates. BMC Cancer.

[13] Makena Frida K., Carole Atieno W.M., Habtu M. (2017). Socio-demographic factors associated with advanced stage of cervical cancer at diagnosis in Kenyatta national hospital, Kenya: a cross sectional study. J. Cancer Sci. Ther..

[14] Sung H., Ferlay J., Siegel R.L., Laversanne M., Soerjomataram I., Jemal A. (2021 May). Global cancer statistics 2020: GLOBOCAN estimates of incidence and mortality worldwide for 36 cancers in 185 countries. CA A Cancer J. Clin..

[15] St-Martin G., Thamsborg L.H., Andersen B., Christensen J., Ejersbo D., Jochumsen K. (2021). Management of low-grade cervical cytology in young women. Cohort study from Denmark. Acta Oncol. (Madr.).

[16] Khuakoonratt N., Tangjitgamol S., Manusirivithaya S., Khunnarong J., Pataradule K., Thavaramara T. (2008). Prevalence of high grade squamous intraepithelial lesion (HSIL) and invasive cervical cancer in patients with low grade squamous intraepithelial lesion (LSIL) at cervical pap smear. Asian Pac. J. Cancer Prev. APJCP.

[17] Jiménez-Wences H., Martínez-Carrillo DiN., Peralta-Zaragoza O., Campos-Viguri G.E., Hernández-Sotelo D., Jiménez-López M.A. (2016). Methylation and expression of miRNAs in precancerous lesions and cervical cancer with HPV16 infection. Oncol. Rep..

[18] Huang P., Zhang S., Li M., Wang J., Ma C., Wang B. (2020). Classification of cervical biopsy images based on LASSO and EL-SVM. IEEE Access.

[19] Friebel-Klingner T.M., Luckett R., Bazzett-Matabele L., Ralefala T.B., Monare B., Nassali M.N. (2021). Clinical and sociodemographic factors associated with late stage cervical cancer diagnosis in Botswana. BMC Wom. Health.

[20] Campos-Romero A., Anderson K.S., Longatto-Filho A., Luna-Ruiz Esparza M.A., Morán-Portela D.J., Castro-Menéndez J.A. (2019). The burden of 14 hr-HPV genotypes in women attending routine cervical cancer screening in 20 states of Mexico: a cross-sectional study. Sci. Rep..

[21] Ryzhov A., Corbex M., Piñeros M., Barchuk A., Andreasyan D., Djanklich S. (2021). Comparison of breast cancer and cervical cancer stage distributions in ten newly independent states of the former Soviet Union: a population-based study. Lancet Oncol..

[22] Shoja Z., Farahmand M., Hosseini N., Jalilvand S. (2019 Oct 1). A meta-analysis on human papillomavirus type distribution among women with cervical neoplasia in the WHO eastern mediterranean region. Intervirology.

[23] Gutié rrez Campos R.I., lica Malacara Rosas A., Gutié rrez Santillá E., Delgado Gutié rrez M., Enrique Torres Orozco R., Daniel García Martínez E. (2019).

[24] Sahin E., Madendag Y., Sahin M.E., Madendag I.C., Acmaz G., Karakukcu C. (2018 Jan 1). Cervical local immune response for high-risk human papillomavirus infection: involvement with cervical mucus SLPI proteins. Cancer Control.

[25] Ekeroma A., Dyer R., Palafox N., Maoate K., Skeen J., Foliaki S. (2019 Sep). Cancer management in the Pacific region: a report on innovation and good practice. The LancetOncology.

[26] Foliaki S., Brewer N., Pearce N., Snijders P.J.F., Meijer C.J.L.M., Waqatakirewa L. (2014). Prevalence of HPV infection and other risk factors in a Fijian population. Infect. Agents Cancer.

[27] Foliaki S. (2011 Mar). Cancer incidence in four Pacific countries : Tonga, Fiji Islands, Cook Islands and Niue. Pac. Health Dialog.

[28] Sarfati D., Dyer R., Sam F.A., Barton M., Bray F., Buadromo E. (2019 Sep). Cancer control in the Pacific: big challenges facing small island states. The LancetOncology.

[29] Law I, Fong JJ, Buadromo EM, Samuela J, Patel MS, Garland SM, et al. The High Burden of Cervical Cancer in Fiji, 2004-2007. 10.1071/SH12135 23557630

[30] Zhang D., Advani S., Huchko M., Braithwaite D. (2020 Jan). Impact of healthcare access and HIV testing on utilisation of cervical cancer screening among US women at high risk of HIV infection: cross-sectional analysis of 2016 BRFSS data. BMJ Open.

[31] Ginsberg G.M., Tan-Torres Edejer T., Lauer J.A., Sepulveda C. (2009). Screening, prevention and treatment of cervical cancer-A global and regional generalized cost-effectiveness analysis. Vaccine.

[32] Law I., Fong J.J., Buadromo E.M., Samuela J., Patel M.S., Garland S.M. (2013). The high burden of cervical cancer in Fiji, 2004-07. Sex. Health.

[33] Wang B., He M., Chao A., Engelgau M.M., Saraiya M., Wang L. (2015). Cervical cancer screening among adult women in China, 2010. Oncol..

[34] Msyamboza K.P., Phiri T., Sichali W., Kwenda W., Kachale F. (2016 Aug). Cervical cancer screening uptake and challenges in Malawi from 2011 to 2015: retrospective cohort study. BMC Publ. Health.

[35] Vodonaivalu L., Bullen C. (2013 Mar). Trends in cervical cancer in Fiji, 2000–2010. Public Health Action.

[36] Anttila A., Ronco G., Clifford G., Bray F., Hakama M., Arbyn M. (2004 Aug). Cervical cancer screening programmes and policies in 18 European countries. Br. J. Cancer.

[37] Obi S.N., Ozumba B.C., Nwokocha A.R., Waboso P.A. (2007 Jan 1). Participation in highly subsidised cervical cancer screening by women in Enugu, South-east Nigeria. J Obstet Gynaecol (Lahore).

[38] Tai Y.J., Chen Y.Y., Hsu H.C., Chiang C.J., You S.L., Chen C.A. (2018 Jul 1). Risks of cervical intraepithelial neoplasia grade 3 or invasive cancers in ASCUS women with different management: a population-based cohort study. J Gynecol Oncol.

[39] Ascus cervical cancer | Mendeley [Internet]. [cited 2021 Oct 29] Available from: https://www.mendeley.com/search/?page=1&query=Ascuscervicalcancer&sortBy=relevance.

[40] Tai Y.J., Chen Y.Y., Hsu H.C., Chiang C.J., You S.L., Chen C.A. (2018). Risks of cervical intraepithelial neoplasia grade 3 or invasive cancers in ASCUS women with different management: a population-based cohort study. J Gynecol Oncol.

[41] Ren C., Zhu Y., Yang L., Zhang X., Liu L., Ren C. (2018 Feb 1). Diagnostic performance of HPV E6/E7 mRNA assay for detection of cervical high-grade intraepithelial neoplasia and cancer among women with ASCUS Papanicolaou smears. Arch. Gynecol. Obstet..

[42] Anttila A., Nieminen P. (2000). Cervical cancer screening programme in Finland. Eur. J. Cancer.

[43] Insinga R.P., Glass A.G., Rush B.B. (2004). Diagnoses and outcomes in cervical cancer screening: a population-based study. Am. J. Obstet. Gynecol..

[44] Rossi P.G., Ricciardi A., Cohet C., Palazzo F., Furnari G., Valle S. (2009 Feb). Epidemiology and costs of cervical cancer screening and cervical dysplasia in Italy. BMC Publ. Health.

[45] Kanyina E.W., Kamau L., Muturi M. (2017). Cervical precancerous changes and selected cervical microbial infections, Kiambu County, Kenya, 2014: a cross sectional study. BMC Infect. Dis..

[46] Maso L.D., Franceschi S., Lise M., Bianchi P.S.D., Polesel J., Ghinelli F. (2010 Jun). Self-reported history of Pap-smear in HIV-positive women in Northern Italy: a cross-sectional study. BMC Cancer.

[47] Padela A.I., Peek M., Johnson-Agbakwu C.E., Hosseinian Z., Curlin F. (2014 Oct). Associations between religion-related factors and cervical cancer screening among Muslims in greater chicago. J. Low. Genit. Tract Dis..

[48] Mukem S., Meng Q., Sriplung H., Tangcharoensathien V. (2016). Low coverage and disparities of breast and cervical cancer screening in Thai women: analysis of national representative household surveys. Asian Pac. J. Cancer Prev. APJCP.

